# Prediction of chronicity in pediatric immune thrombocytopenia based on developed and internally validated machine learning models

**DOI:** 10.3389/fped.2026.1908774

**Published:** 2026-08-19

**Authors:** Xiaoqin Zhang, Benshan Zhang, Wanli Li, Yang Liu, Huimin Xu, Zhijun Huang, Wenyong Kuang

**Affiliations:** Department of Hematology, The Affiliated Children’s Hospital of Xiangya School of Medicine, Central South University (Hunan Children’s Hospital), Changsha, Hunan, China

**Keywords:** chronicity prediction, ET model, machine learning, platelet-specific antibodies, primary immune thrombocytopenia

## Abstract

**Background:**

Primary immune thrombocytopenia (ITP) typically remits spontaneously, 20%–30% of children progress to chronic ITP. Early prediction of chronicity in ITP may facilitate in timely intervention and improve prognosis.

**Methods:**

In our current study, we collected clinical features such as platelet-specific antibodies and used machine learning (ML) to predict chronic progression of ITP. ML models were applied to data from our hospital. Model performance was evaluated using accuracy, precision, sensitivity, specificity, *F*1 score, and area under the receiver operating characteristic (ROC) curve to assess the binary classification models performance.

**Results:**

A total of 156 patients were enrolled for ITP classification prediction model construction, including 68 chronic patients and 88 non-chronic patients. All models performed well, with AUC values ranging from 0.833 to 0.864. The ET model was selected for predictive model construction due to its highest AUROC score and interpretability. Then, the ET model identified occult disease course, age, and platelet-specific antibodies as significant predictors. The absence of an occult disease course decreased the probability of chronic ITP, while older age increased it. It's worth noting when platelet-specific antibodies are negative, patients are less likely to develop chronic ITP.

**Conclusions:**

In conclusion, the ET model achieved high prediction accuracy for ITP chronicity by using clinical parameters, especially platelet-specific antibodies. Despite the limited sample size, this study suggests occult disease course, age, and platelet-specific antibodies are early predictors of chronic ITP in children, meriting confirmed in future larger cohort multicenter study.

## 1 Introduction

Primary immune thrombocytopenia (ITP) is a autoimmune bleeding disorder in children, with an incidence of 8.4–14.3 per 100,000 (1, 2). A meta-analysis by Shen et al. indicated that the global burden of hematologic malignancies remained heavy in recent years (3). Childhood ITP presents with thrombocytopenia and a range of bleeding symptoms, although major bleeding events, including intracranial hemorrhage, are infrequent (4, 5). Approximately 20%–30% of ITP patients develop chronic ITP, which reduces quality of life (6, 7). Predicting the likelihood of chronicity in ITP patients aids in early detection and timely intervention, thus improving disease prognosis.

Advanced age is an established risk factor for chronic ITP development (5, 8). The absence of triggering factors or mucosal bleeding, elevated platelet count, and positive direct antiglobulin test at diagnosis predict chronic ITP development (9, 10). While reliable predictors of chronicity are still lacking at the time of ITP diagnosis, the disease pathophysiology involves both humoral and cellular immune responses targeting platelet self-antigens (11, 12). Thus, platelet-specific antibodies testing could potentially be used in the diagnosis and determination of prognosis of ITP. However, the role of platelet-specific antibodies testing in predicting chronicity at the time of ITP diagnosis remain largely unknown.

Machine learning (ML), a branch of artificial intelligence in computer science, uses statistical methods to enable computer systems to “learn” from data and perform specific tasks without being explicitly programmed (13). ML has proven highly effective in modeling extensive datasets and detecting intricate nonlinear patterns. Although ML has shown promise in various medical applications, its application in predicting chronic disease progression in children with ITP remains limited. To address this gap, this current study developed and internally validated six machine learning models that integrated demographic and clinical features to predict chronic ITP. This study utilized the Extra-Trees algorithm, an extension of the random forest technique (14). Unlike random forests, which use bootstrap samples to determine node splits, the Extra-Trees algorithm operates by randomly selecting features and split thresholds from the entire training dataset, which boosts diversity and lowers variance (15, 16). Through this approach, we aimed to develop and internally validate six machine learning models that integrated demographics and characteristics to predict the relationship between platelet-specific antibodies and the chronicity of ITP.

## 2 Methods

### 2.1 Study design and patient enrollment

In this retrospective study, we trained and internally validated ML algorithms to predict the chronicity of newly diagnosed pediatric ITP using demographic and immunologic features extracted from electronic medical records of patients at The Affiliated Children's Hospital of Xiangya School of Medicine, Central South University, between August 2023 and June 2024. This study enrolled pediatric patients (aged 1 month–16 years) with newly diagnosed primary ITP (<3 months from diagnosis) and no evidence of secondary causes. The non-chronic group also includes patients with relapsed but non-chronic ITP (disease duration not exceeding 12 months). Participants were treatment-naïve at enrollment and had 12-month follow-up data. Exclusion criteria comprised: (1) receipt of immunomodulators (e.g., IVIG, corticosteroids) within 3 months post-diagnosis, or (2) active infection at admission. To mitigate confounding effects of early immunomodulatory therapies-which may alter immune markers-we exclusively included children who received no immunotherapy prior to biomarker analysis.

### 2.2 Data collection

Demographic, clinical, and immunologic data were extracted from electronic medical records. Variables included age, sex, disease duration, precipitating factors, bleeding manifestations, bone marrow smear megakaryocyte count, platelet count, platelet volume, lymphocyte count, lymphocyte ratio, blood type, immunoglobulin A, immunoglobulin E, immunoglobulin G, immunoglobulin M, complement C3, and complement C4. Platelet-specific antibodies were also included in the analysis. During the 12-month follow-up period, patients were interviewed personally in the clinic or by telephone to assess duration of disease remission or chronicity, bleeding severity, and platelet-specific antibodies.

### 2.3 Definition of chronicity of ITP

Our study adopted the 2019 American Society of Hematology guideline (17), which defines chronic ITP as disease persistence for at least 12 months. Accordingly, we considered ITP to be chronic when thrombocytopenia persisted beyond a follow-up period of 12 months in patients.

### 2.4 ML development process

#### 2.4.1 Data preprocessing

We employed a dataset of 156 cases to build and evaluate the ITP prediction model. The dataset was randomly partitioned, with 70% of the data assigned to the training dataset and the remaining 30% to the test dataset. Model training, parameter tuning, and cross-validation were all performed on the training dataset, while the test dataset was used solely to evaluate the model's classification performance. Stratified sampling was used to partition the dataset, ensuring that the proportions of each data type in the training and internal test datasets remained consistent with those in the original data. The data were then normalized, first on the training dataset and then applied to the test dataset, ensuring the relative independence of the test set data.

#### 2.4.2 Development of the models

This study uses six machine learning models to complete the classification task: logistic regression (LR) (18), support vector machine (SVM) (19), random forest (RF) (20), gradient boosting decision trees (GBDT) (21), adaptive boosting (ADA) (22), and extreme random trees (ET) (23). In the training phase, the optimal parameter configuration for each model was identified through a grid search coupled with ten-fold cross-validation. This optimized configuration was subsequently applied to the test dataset to evaluate the model's classification performance. For the LR model, the parameters adjusted include the regularization type (L1/L2), the regularization strength C, and the optimization algorithm (solver). For the SVM model, the parameters adjusted include the regularization parameter C, the kernel function type rbf, and the parameter controlling the kernel function (gamma). For the RF model, the parameters to be adjusted include the number of decision trees (n_estimators), the maximum tree depth (max_depth), the minimum number of samples for leaf nodes (min_samples_leaf), and the minimum number of samples for internal node splits (min_samples_split). For the GBDT model, the parameters to be adjusted include the learning rate (learning_rate), the number of decision trees (n_estimators), the maximum tree depth (max_depth), and the minimum number of samples for internal node splits (min_samples_split). For the ADA model, the parameters to be adjusted include the learning rate (learning_rate) and the number of decision trees (n_estimators). For the ET model, the parameters to be adjusted include the number of decision trees (n_estimators), the number of features considered by each tree (max_features), the minimum number of samples for leaf nodes (min_samples_leaf), and the minimum number of samples for internal node splits (min_samples_split).

#### 2.4.3 Evaluation and validation of the models

Overall, the performance of binary classification models is evaluated using accuracy, precision, sensitivity, specificity, *F*1 score, and the area under the receiver operating characteristic (ROC) curve. Accuracy represents the proportion of correctly predicted samples to the total number of samples (Equation 1). Precision represents the proportion of correctly predicted samples to the total number of positive predictions (Equation 2). Sensitivity represents the proportion of correctly predicted samples to the total number of actual positive samples (Equation 3). Specificity represents the proportion of actual negative samples that were incorrectly predicted as positive (Equation 4). The *F*1 score measures the accuracy and sensitivity of a classification model (Equation 5). The ROC curve plots the false positive rate (false positive rate) against the true positive rate (true positive rate) at different thresholds. A larger area under the curve (AUC) indicates better classification performance. TP, FP, TN, and FN represent the number of positive samples predicted as positive, the number of negative samples predicted as positive, the number of negative samples predicted as negative, and the number of positive samples predicted as negative, respectively. All model building and analysis processes were implemented on Python 3.9.

$$accuracy=\frac{TP+TN}{\left(TP+FP+TN+FN\right)}$$ (1)

$$precision=\frac{TP}{\left(TP+FP\right)}$$ (2)

$$sensitivity=\frac{TP}{\left(TP+FN\right)}=recall$$ (3)

$$specificity=\frac{TP}{\left(FP+TN\right)}$$ (4)

$$F1-score=\frac{2*(precision*recal)}{\left(precision+recal\right)}$$ (5)

### 2.5 Statistical analysis

Continuous data were analyzed using Mann–Whitney *U* test and are presented as mean ± SD. Categorical data were assessed using either the chi-squared test or Fisher's exact test, with results reported as number and percentage. A 2-tailed *P* value of <0.05 was considered statistically significant.

## 3 Results

### 3.1 Construction of ITP classification prediction model and baseline characteristics

In this study, a classification and prediction model for ITP was constructed using a sample of 156 patients, including 68 in the chronic group and 88 in the non-chronic group. In addition, 18 clinical features were collected as model inputs. A general description of these features is shown in Table 1. Our results indicated that the age of the chronic group was significantly higher than that of the non-chronic group (*P* < 0.001). Furthermore, the chronic group had a more frequent occult disease course compared to the non-chronic group (*P* < 0.001). Occult disease course refers to the presence of bleeding symptoms or thrombocytopenia lasting more than 2 weeks before the diagnosis of ITP. Recent studies show that occult disease course may be a risk factor for chronic ITP (9, 24). Feature numbers are counted starting at 0 by default.

**Table 1:** General case of input features.

Feature number	Contents		Chronic (*n* = 68)	Non-chronic (*n* = 88)	*P* value
0	Age (years)	≤1	4 (5.9)	21 (23.9)	<0.001
		1–6	25 (36.8)	45 (51.1)	
		6–10	15 (22.1)	16 (18.2)	
		>10	24 (35.3)	6 (6.8)	
1	Gender	Male	37 (54.4)	55 (62.5)	0.308
		Female	31 (45.6)	33 (37.5)	
2	Hidden course	Yes	41 (60.3)	21 (23.9)	<0.001
		No	27 (39.7)	67 (76.1)	
3	Predisposing factors	Yes	20 (23.1)	33 (37.5)	0.290
		No	48 (44.9)	55 (62.5)	
4	Bleeding manifestations	Dry	45 (66.2)	60 (68.2)	0.754
		Wet	20 (29.4)	26 (29.5)	
		None	3 (4.4)	2 (2.3)	
5	Bone marrow smear megakaryocyte count		156.74 ± 114.97	165.34 ± 83.19	0.297
6	Platelet count		16.54 ± 14.56	14.73 ± 11.73	0.461
7	Platelet volume		12.8 ± 9.69	14.51 ± 14.43	0.512
8	Lymphocyte count		3.28 ± 1.83	4.13 ± 1.95	0.002
9	Lymphocyte ratio		0.41 ± 0.18	0.51 ± 0.16	0.001
10	Blood type	A	26 (38.2)	34 (38.6)	0.378
		B	20 (29.4)	22 (25)	
		AB	1 (1.5)	6 (6.8)	
		O	21 (30.9)	26 (29.5)	
11	Immunoglobulin A		1.57 ± 2.30	0.89 ± 0.67	0.002
12	Immunoglobulin E		206.38 ± 299.97	89.49 ± 167.12	0.014
13	Immunoglobulin G		15.44 ± 9.42	10.70 ± 6.05	0.001
14	Immunoglobulin M		1.21 ± 0.52	1.08 ± 0.52	0.140
15	Complement C3		0.90 ± 0.20	0.85 ± 0.17	0.369
16	Complement C4		0.19 ± 0.07	0.21 ± 0.12	0.158
17	Platelet-specific antibodies	Anti-GPIIb/IIIa antibody positive only	3 (4.4)	5 (5.7)	0.016
		Anti-GPIb/IX antibody positive only	7 (10.3)	1 (1.1)	
		Double antibody positive	11 (16.2)	7 (8)	
		Double antibody negative	47 (69.1)	75 (85.2)	

### 3.2 Evaluation of model classification performance

**Figure 1:**
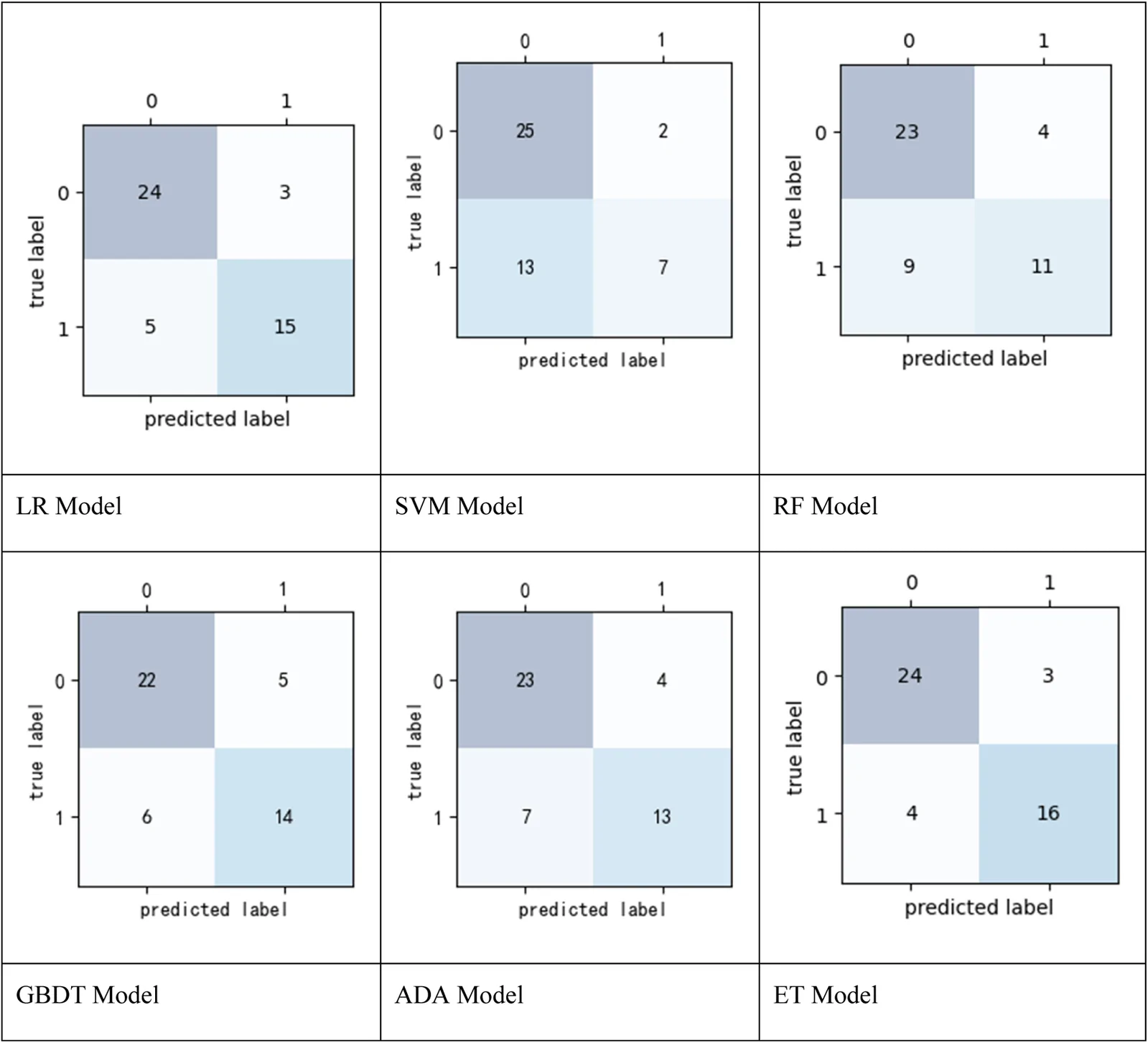
Confusion matrix of different machine learning models.

**Figure 2:**
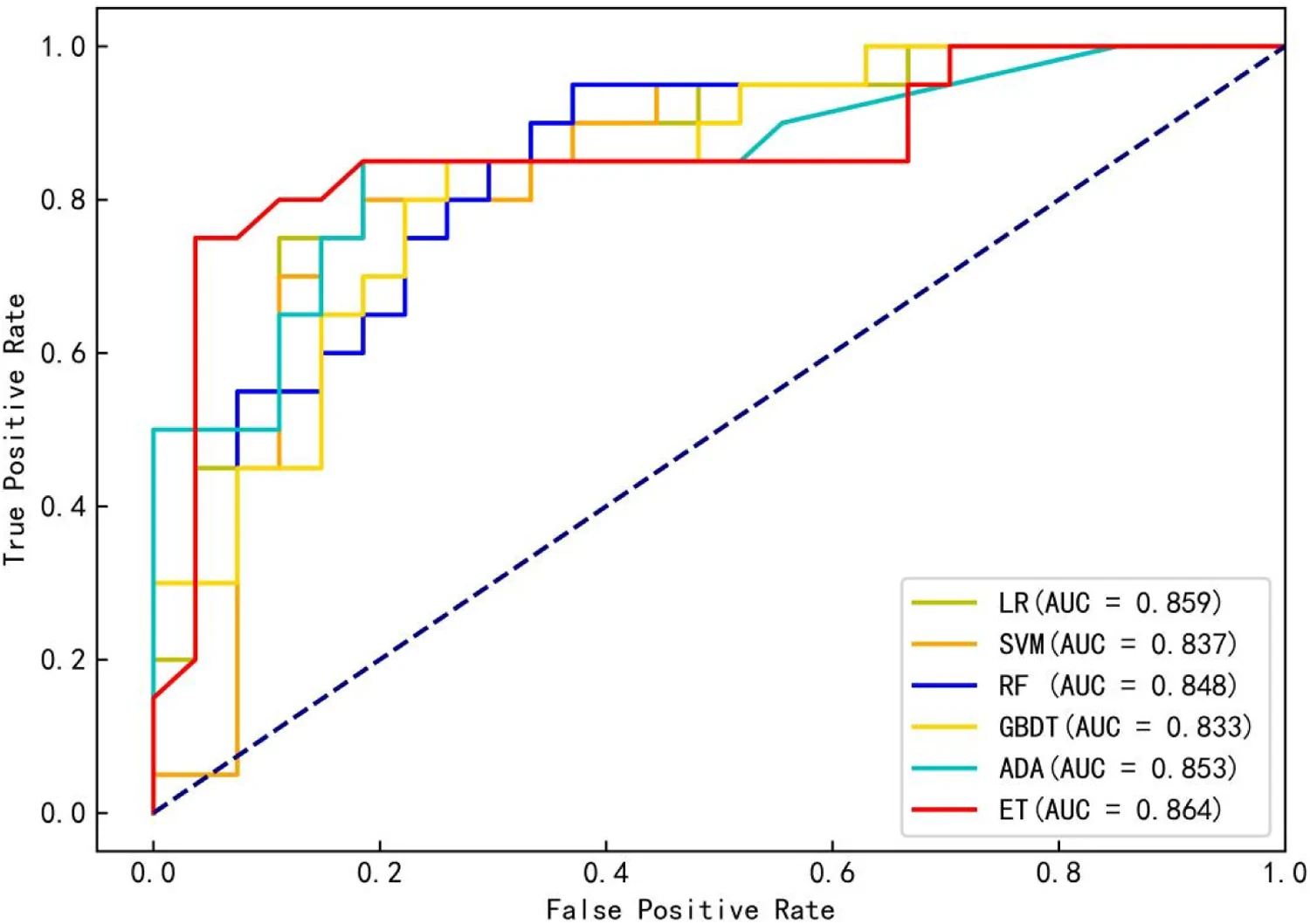
ROC curves of different machine learning models.

To enhance the discrimination between chronic and non-chronic ITP, six widely-used machine learning models were developed and compared on a common test set in this study. Figure 1 displays the confusion matrices for each model, where the lower-right quadrant represents true positives (i.e., chronic ITP correctly classified) and the upper-left quadrant indicates true negatives (non-chronic ITP accurately identified). Based on these matrices, standard performance metrics for each model were computed and summarized in Table 2. Among the models, the SVM achieved high specificity (0.926), reflecting a strong ability to identify non-chchronic cases, yet it demonstrated comparatively lower overall accuracy and sensitivity. In contrast, the ET model outperformed others across multiple metrics—including accuracy, precision, sensitivity, and *F*1-score—indicating superior classification capability. ROC analysis (Figure 2) revealed that all models attained satisfactory discriminative power, with AUC values ranging from 0.833 to 0.864. Notably, the ET model achieved the highest AUC, consistent with its overall excellence in classification performance.

**Table 2:** Evaluating the classification capabilities of different ML models.

Model	Accuracy	Precision	Sensitivity	Specificity	F1
LR	0.830	0.833	0.750	0.889	0.789
SVM	0.681	0.778	0.350	**0.926**	0.483
RF	0.723	0.733	0.550	0.852	0.629
GBDT	0.766	0.737	0.700	0.815	0.718
ADA	0.766	0.765	0.650	0.852	0.703
ET	**0.851**	**0.842**	**0.800**	0.889	**0.821**

The bolded numbers indicate that its classification ability is the highest.

**Figure 3:**
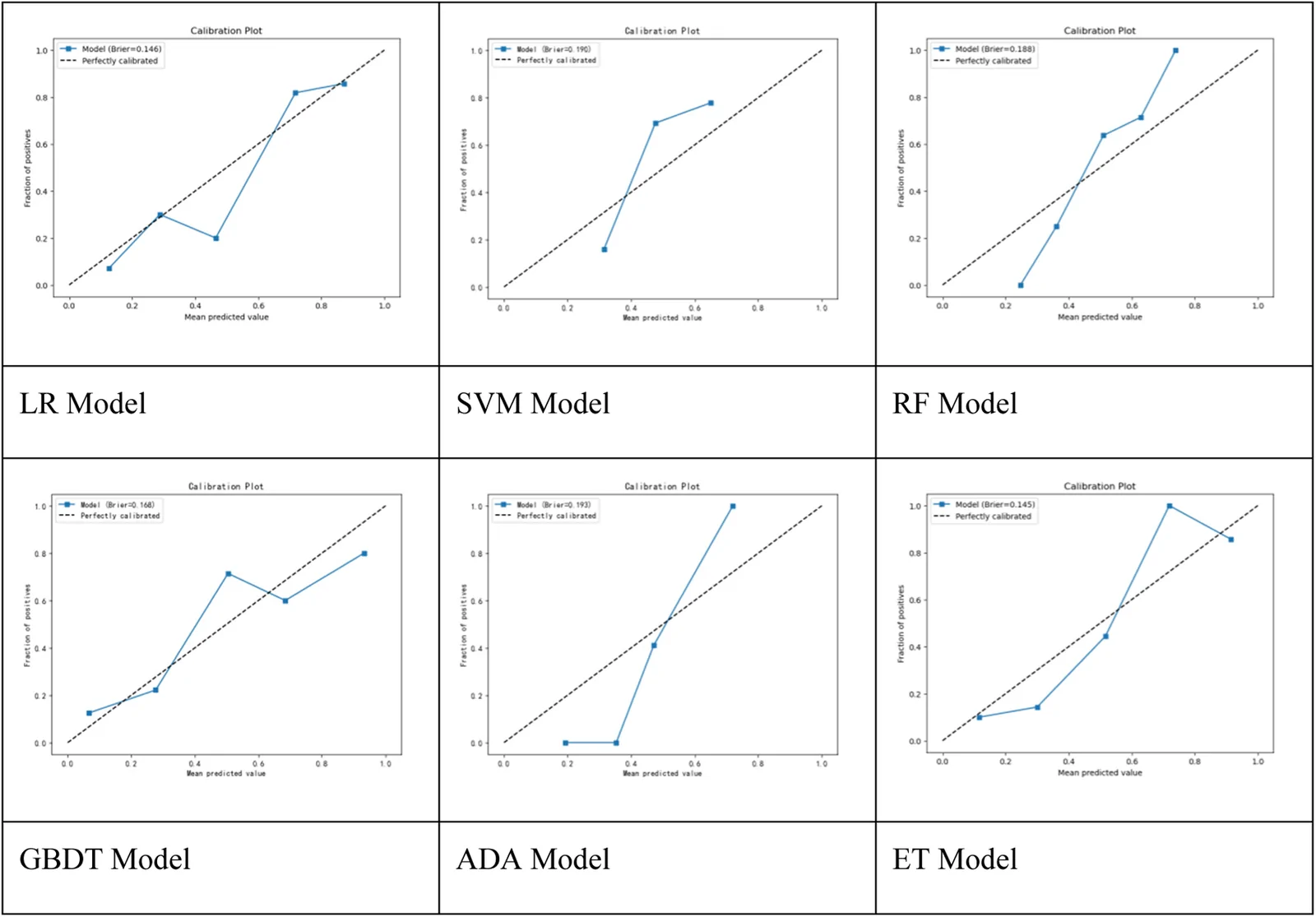
The calibration curves of six machine learning models.

**Figure 4:**
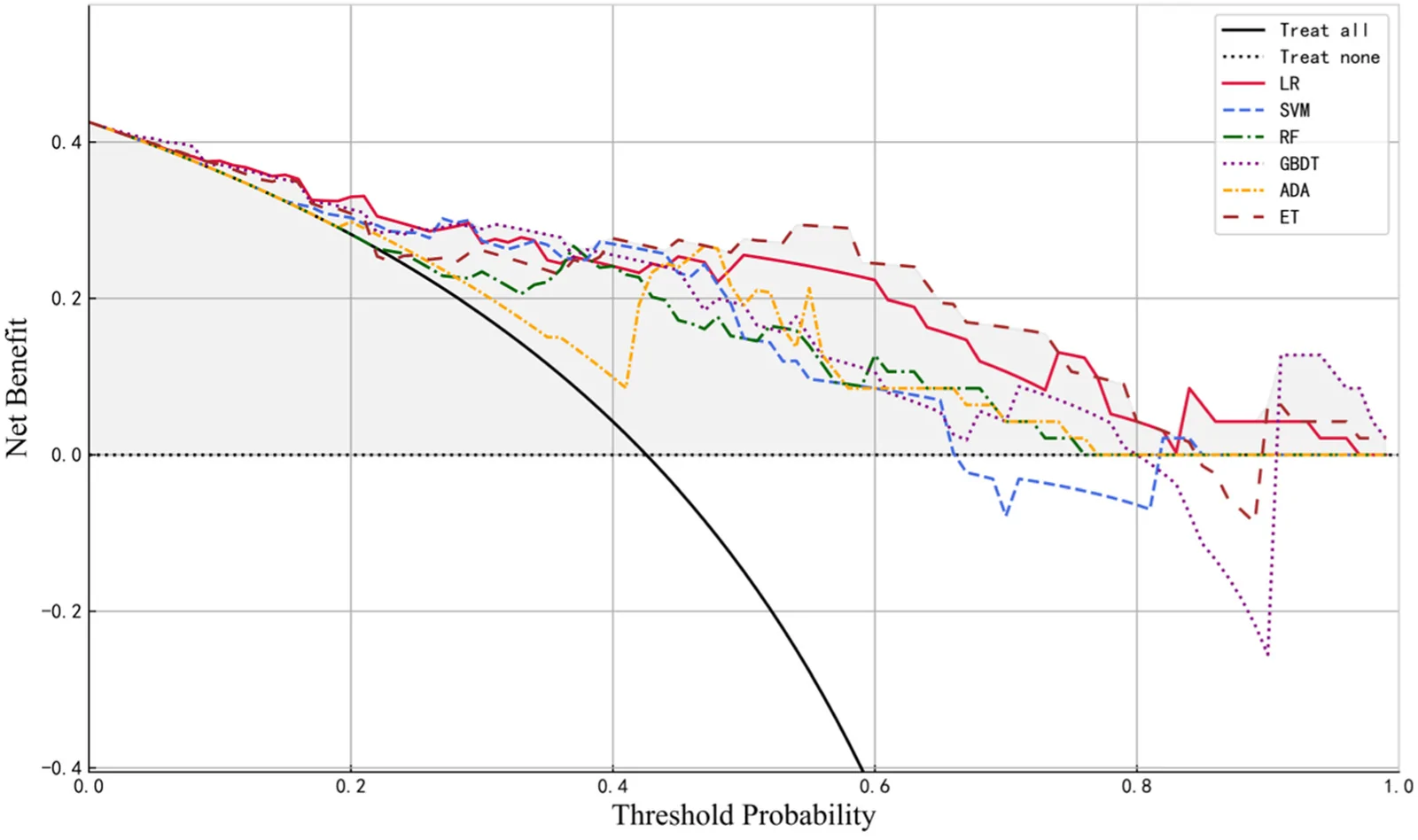
Decision curves of different machine learning models.

Figure 3 presents the calibration curves for the six machine learning models. The *x*-axis denotes the mean predicted probability, while the *y*-axis represents the actual observed frequency. A curve closely aligned with the diagonal reference line (*y* = *x*) reflects well-calibrated predictions, indicating close agreement between predicted probabilities and observed outcomes. The results demonstrate that all models showed good calibration across a relevant probability range, with curves approximating the diagonal. Furthermore, the Brier scores for all models ranged from 0.145 to 0.193, with the ET model and LR model achieving Brier scores of 0.145 and 0.146, respectively, indicating that these two models have superior reliability in probabilistic prediction. Decision curves quantify the net benefit at different intervention thresholds, helping decision makers assess the practical value of model predictions. As shown in Figure 4, the decision curves for all models remained within the Treat All and Treat None curves, indicating that the models have clinical utility within a certain range of probability thresholds. The ET, LR, and GBDT models all demonstrated benefit in assisting clinical decision making within the threshold range of 0.3–0.8. In summary, the ET model performed best among the six machine learning models, with accuracy, sensitivity, specificity and AUC values of 0.851, 0.800, 0.889 and 0.864, respectively. The LR model performed second best.

### 3.3 Analysis of contributions of clinical feature

**Figure 5:**
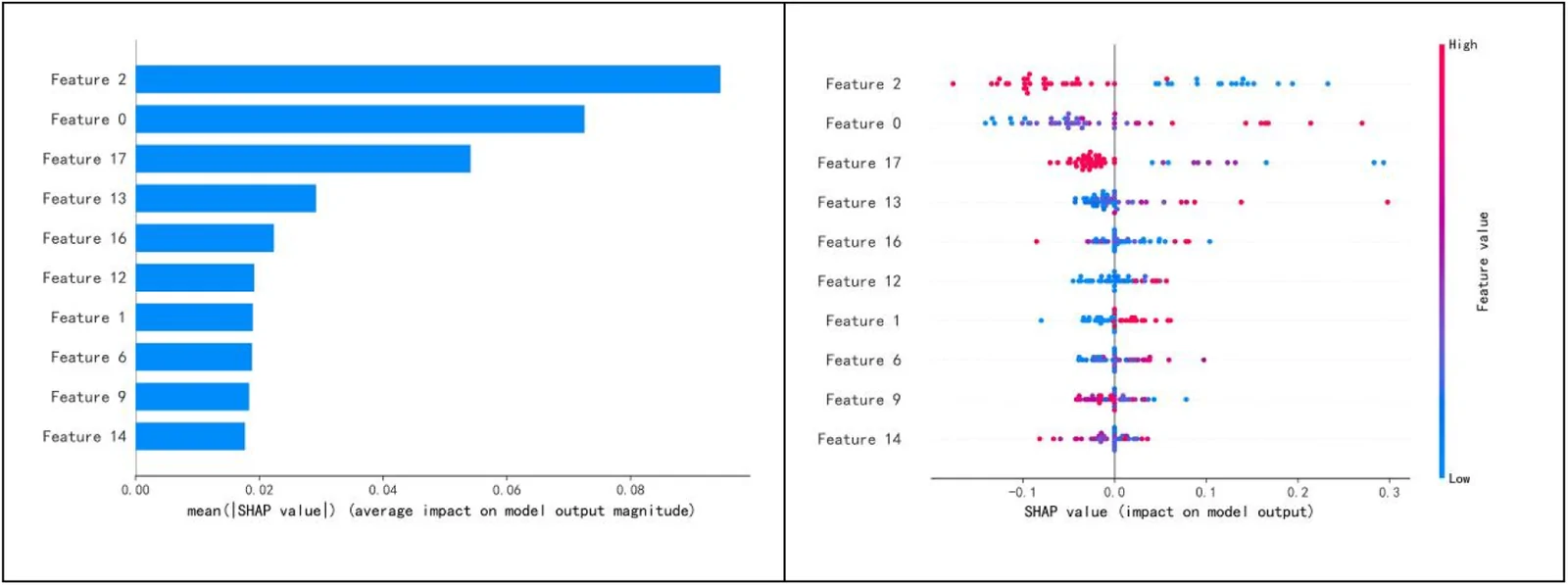
Importance analysis of the first ten features of the ET model.

For the higher-performing ET models, SHapley Additive exPlanations (SHAP) was used to perform an importance analysis of the input features (only the top ten important features are shown), as shown in Figure 5. The left figure shows the feature importance ranking generated based on SHAP values. The higher the average SHAP value of a feature, the more important it is in the model. The right figure shows a global feature influence map based on the SHAP values. The SHAP value reflects the degree of influence of a feature on the model output. In this map, each feature's value is represented by a different colored dot, with red indicating a higher value and blue indicating a lower value. This map not only provides a ranking of feature importance but also provides an intuitive view of the overall distribution of each feature's impact on the model output. The results suggest that the top three important features are occult disease course, age, and platelet-specific antibodies. For occult disease course, the vast majority of red dots are located in the negative SHAP value region, indicating that the absence of an occult disease course negatively impacts the model output and reduces the probability of chronic ITP in patients. For the age feature, the vast majority of red points are distributed in the positive SHAP value area, indicating that older age increases the probability of chronic ITP. For platelet-specific antibodies, the vast majority of red points are distributed in the negative SHAP value area, indicating that negative platelet-specific antibodies have a negative impact on the model output and reduce the probability of chronic ITP in patients.

## 4 Discussion

Chronic ITP remains relatively uncommon in children, which warrants particular clinical attention (25). Early and accurate prediction of disease chronicity is crucial due to its link to potential treatment-related adverse effects. Despite this importance, a standard predictive framework for pediatric ITP remains absent. A prospective cohort study utilizing the Intercontinental Collaborative ITP Study Group Registry II identified age under one year and moderate to severe bleeding as predictors of chronic disease, but found no significant correlation with gender or baseline platelet count (26). In this research, we compared the clinical characteristics of children with ITP patients yielded results consistent with previous studies and initial findings from our hospital.

ITP is an antibody-mediated autoimmune disorder (27). This disease primarily involves autoantibodies against platelet antigen complexes, most commonly platelet glycoproteins GPIIb/IIIa and GPIb/IX. These autoantibodies are thought to accelerate platelet destruction by the reticuloendothelial system while inhibiting megakaryocyte formation (6, 28, 29). Studies in adult ITP patients have shown that different platelet-specific antibodies can influence clinical progression, with the presence of anti-GPIb/IX autoantibodies being a predictor of poor prognosis (30). Fabrizio et al. reported that patients carrying anti-GPIb/IX antibodies or antibodies against multiple antigens experienced more severe bleeding symptoms (31). Autoantibodies reacting with glycoproteins (GP) IIb/IIIa may affect platelet aggregation; these are the most common autoantibodies in primary ITP. Anti-GPIb/IX antibodies may disrupt platelet adhesion to the subendothelial matrix, leading to severe bleeding (32). However, this association has not been investigated in pediatric ITP patients. The pathophysiology of ITP involves humoral and cellular immune responses against platelet autoantigens (6, 33). Therefore, platelet-specific antibodies detection may be helpful for the diagnosis and prognosis assessment of ITP. However, the role of platelet-specific antibodies detection in predicting the progression of ITP to the chronic stage at the initial diagnosis stage remains unclear. In our study, we performed platelet-specific antibodies testing and stratified patients into the following groups: Group 1: GP IIb/IIIa antibody positive; Group 2: GPIb/IX antibody positive; Group 3: Both antibodies positive; and Group 4: Both antibodies negative. The results showed that the proportion of patients with both antibodies negative was 78.2%.

Given that ITP prognosis is influenced by multiple interacting variables with complex nonlinear relationships, a statistically significant difference in a single factor between groups does not fully clarify its prognostic value, especially when interactions with other variables remain unknown. Relying solely on univariate analysis for prognostic prediction is therefore inadequate. To address these limitations, we applied ML techniques to further investigate the multifactorial influences on predicting the chronicity of ITP. ML offers distinct advantages over conventional statistical methods by holistically assessing the collective impact of variables and effectively modeling complex nonlinear relationships (34).

In the present study, we developed six machine learning models to identify chronic and non-chronic ITP. Among the six models, the ET model performed best in accuracy, precision, sensitivity, and *F*1 score, demonstrating better classification capabilities. Furthermore, the ET model had the highest AUC value and exhibited superior overall performance across all evaluation metrics involved. Within a specific range, the model calibration curve closely aligned with the diagonal line, indicating good model calibration. The Brier score of the ET model also demonstrated excellent probabilistic predictive reliability. Overall, the ET model performed best among the six machine learning models, with accuracy, sensitivity, specificity. Consequently, we selected the ET model as our 1-year chronicity prediction model. In addition, we have some interest in the broader field of AI-assisted clinical prediction and have consulted the latest methodological discussions on this topic, which may be applied in our future research (35, 36).

SHAP was then employed to analyze the importance of input features for the better-performing ET models. The higher the average SHAP value of a feature, the more important it is in the model. The results of this study showed that the top three important features were occult disease course, age, and platelet-specific antibodies. The absence of an occult disease course reduces the probability of chronic ITP. Older age increases the probability of chronic ITP, which has also been confirmed in previous studies. Notably, when platelet-specific antibodies are negative, the probability of chronic ITP is lower, which is also a relatively clear and detectable indicator. Fu et al. also found 3 of 4 patients with anti-GPIb/IX developed chronic ITP (37). Furthermore, the clinical significance of using antibody testing as a screening tool in routine pediatric care warrants further exploration in our future research, perhaps by learning how biomarker-based screening tools are evaluated in other disease areas (38, 39). Additionally, we did not collect data on patients’ history of autoimmune diseases or immunodeficiency in the present study, and this should be addressed in future research.

In the current study, the machine learning model we developed could serve as a valuable screening tool for predicting the chronic stage in children with ITP, thereby assisting clinical decision-making at an early stage of the disease. The ET model presented acceptable predictive performance and high accuracy was developed to identify patients at risk of chronic disease. Furthermore, SHAP analysis identified occult disease course, age, and platelet-specific antibodies as the three most important features in the model. Of particular note, negative platelet-specific antibodies were associated with a significantly lower probability of chronic ITP, highlighting their potential utility as a critical predictor of chronic ITP.

## Data Availability

The datasets presented in this study can be found in online repositories. The names of the repository/repositories and accession number(s) can be found in the article/Supplementary Material.
